# An update on the distribution and nomenclature of fleas (Order Siphonaptera) of bats (Order Chiroptera) and rodents (Order Rodentia) from La Rioja Province, Argentina

**DOI:** 10.3897/zookeys.678.12006

**Published:** 2017-06-07

**Authors:** M. Fernanda López Berrizbeitia, R. Tatiana Sánchez, Ruben M. Barquez, M. Monica Díaz

**Affiliations:** 1 PIDBA (Programa de Investigaciones de Biodiversidad Argentina) – PCMA (Programa de Conservación de los Murciélagos de Argentina), CONICET (Consejo Nacional de Investigaciones Científicas y Técnicas), Facultad de Ciencias Naturales e Instituto Miguel Lillo, Universidad Nacional de Tucumán, Miguel Lillo 205, (4000) Tucumán, Argentina; 2 Fundación Miguel Lillo; 3 Centro Regional de Investigaciones Científicas y Transferencia Tecnológica (CRILAR- CONICET)

**Keywords:** Distribution map, mammals, new records, northwestern Argentina, Siphonaptera

## Abstract

The mammalian and flea fauna of La Rioja Province is one of the least known from northwestern Argentina. In this study, the distribution and nomenclature of 13 species of fleas of bats and rodents from La Rioja Province are updated. Four species of fleas are recorded for the first time in La Rioja Province including a new record for northwestern Argentina, and two new flea-host associations. An identification key and distribution map are included for all known species of Siphonaptera of bats and rodents from La Rioja Province, Argentina.

## Introduction

The flea fauna of the northern province of La Rioja (Argentina) has received little attention historically and accounts for the least number of documented flea records of any province in Argentina. Four different ecoregions are represented in La Rioja: High Andes, Puna, Dry Chaco, and Monte Desert of Mountains and Isolated Valleys (Burkart et al. 1999), all belonging to the arid and semi-arid environments of Argentina, containing a number of little known mammal species (Ojeda et al. 1998, Barquez et al. 2006). Ectoparasites, including fleas, display different degrees of host specificity from generalists to specialists (Lareschi et al. 2004; Poulin et al. 2006). It is important to note that unexplored geographic areas, containing species of little known small mammals, represent an interesting source for the discovery of new parasites for the study area, extensions of their distributions, and even the discovery of forms new to science.

In this study, an update on the distribution and nomenclature of the fleas of bats and rodents from La Rioja Province is offered, including new records of fleas for the province, a new record for northwestern Argentina, and additional new flea-host associations.

## Materials and methods

Fleas were collected from several sites in different ecoregions: Puna, Dry Chaco, and Monte Desert of Mountains and Isolated Valleys of La Rioja Province. Surface-dwelling mammals were captured with Sherman live traps baited with oats and set in transects; fossorial mammals were collected with traps designed for the live capture of subterranean rodents, modified from the model of Baker and Williams (1972), placed early in the morning at the entrance of active burrows, and checked every two hours. Mist nets were employed from sunset until midnight to capture bats. Fleas were removed from each mammal specimen with forceps and placed in vials filled with ethanol 70%. Fleas were mounted on microscope slides in accordance with conventional procedures that included puncturing the area between abdominal sterna II and III with a minute pin, soaking for 24 h in potassium hydroxide (10%), transferring to distilled water and gently compressing the flea’s abdomen to expel macerated soft tissues, dehydration in a series of ethanol solutions (70%, 80%, 95%, absolute) for 30 minutes each, clarifying the exoskeleton for 15–20 minutes in methyl salicylate, transferring to xylene for a minimum of 1 h, and mounting in Canada balsam (see Hastriter and Whiting 2003).

**Figure 1.**
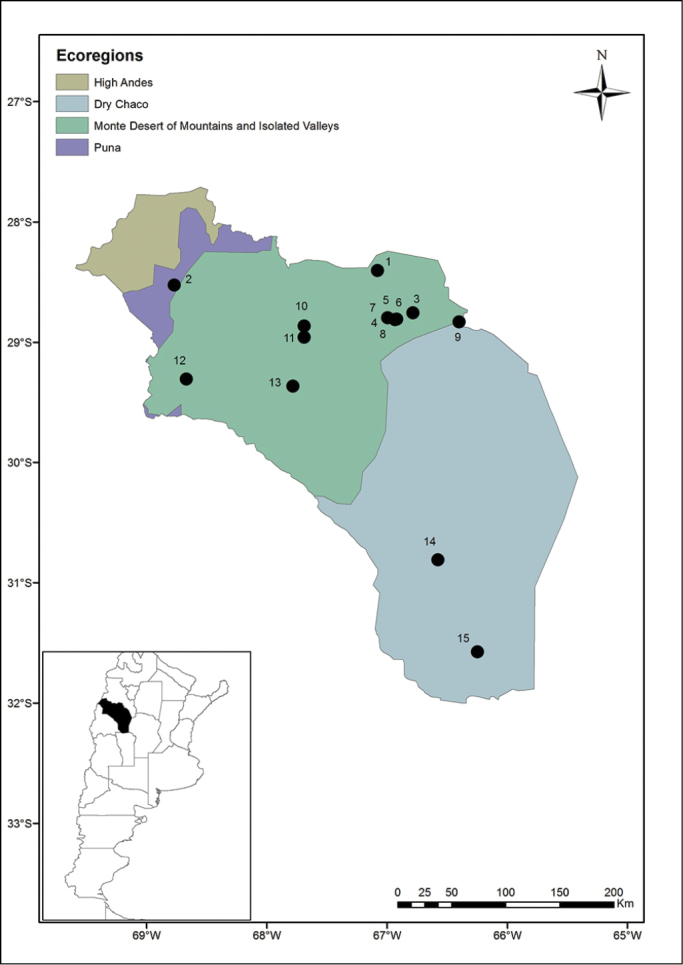
Map illustrating the localities of fleas of bats and rodents from La Rioja Province, Argentina. The symbol with associated locality number are listed on map by latitude from north to south. The localities include specific locality, coordinates, department, and altitudes as follow: **1** 700 m E of National Route 40 (28°24'17.4"S, 67°04'48.4"W), San Blas Department, 1123 m **2** Quebrada de Santo Domingo 30 km SW of Jagüé (28°31'34.7"S, 68°46'13.8"W), Vinchina Department, 3131 m **3** 2 km S Río de La Punta on provincial road 7 (28°45'28.8"S; 66°47'09.3"W), Arauco Department, 996 m **4** Reserva Aguada de las Alturas, 4 km W Anillaco (28°47.942'S, 66°59.749'W), Castro Barros Department, 1188 m **5** Anillaco, 500 m W of plaza de Anillaco (28°48'40.30"S, 66°55'55.55"W), Castro Barros Department, 500 m **6** Anillaco 1.7 m E of CRILAR (28°48'46.00"S, 66°55'50.44"W), Castro Barros Department, 1357 m **7** Anillaco, behind cemetery (28°48'49.04"S, 66°56'0.75"W), Castro Barros Department, 1365 m **8** 800 m E Anillaco (28°48.572’ S; 66°55.193’ W), Castro Barros Department, 780 m **9** Cuesta La Cébila, 22 km NW of Chumbicha, by route 60 (28°50'S, 66°24'W), Capital Department: 1066 m **10** 5 km S of Cañón del Ocre (28°51'55.9"S, 67°41'26.3"W), Famatina Department, 2495 m **11** 8 km NE of Cañón del Ocre, (28°57'37.3"S, 67°41'26.3"W), Famatina Department, 3127 m **12** Zapallar, (29°18'24.74"S, 68°40'9.2"W), Coronel Felipe Varela Department, 1634 m **13** 1 km N Los Tambillos (29°22'S, 67°47'W), Coronel Felipe Varela Department, 1951 m **14** 2 km E of Malanzán, camping El Descanso (30°48'37.7’”S, 66°34'40.3"W), General Facundo J. Quiroga Department, 957 m **15** Ulapes, 1 km W of plaza principal de Ulapes (31°34'35"S, 66°14'55"W), San Martín Department, 493 m.

The images were prepared using a Leica M205A stereo microscope with a Leica DFC295 camera supported by Leica Application Suite Version 4.8.0. The distribution map was designed with an ArcGis 10.1 program (ESRI, 2011). ArcGIS Desktop: Release 10. Redlands, California: Environmental Systems Research Institute. The map (Fig. 1) shows the localities mentioned in the text; the numbers are indicated in brackets in front of each locality in Material Examined and Additional Records.

Mammalian nomenclature follows that of Wilson and Reeder (2005), Gardner (2008), Coyner et al. (2013), and Patton et al. (2015). Some mammals not yet identified at the species level are cited as sp. since they are under study. Fleas were identified using keys and descriptions by Hopkins and Rothschild (1953, 1956), Johnson (1957), Smit (1987), Hastriter and Mendez (2000), Lareschi and Linardi (2009), Sanchez et al. (2012) and López Berrizbeitia et al. (2015). The classification of Siphonaptera is based on Whiting et al. (2008). Voucher specimens of hosts are deposited in the Colección Mamíferos Lillo (CML), Universidad Nacional de Tucumán, Argentina. Some of the host specimens are still being catalogued for the CML Collection; for this reason, the acronym used in the text corresponds to the initials of the collector, Rocío Tatiana Sánchez (RTS). Fleas are deposited in the Colección Mamíferos Lillo “Anexos” (CMLA), Universidad Nacional de Tucumán, Argentina.

## Results

### Family Tungidae

#### Subfamily Tunginae

##### Hectopsylla (Hectopsylla) cypha

Taxon classificationAnimaliaSiphonapteraTungidae

Jordan

###### Distribution in Argentina.

La Rioja, Mendoza, Río Negro, and Tucumán (Lareschi et al. 2016).

###### Material examined.

None.

###### Additional records.

Coronel Felipe Varela Department: (12) Zapallar (29°18'24.74"S, 68°40'9.2"W), 1634 m, *Lagostomus* sp., 1 ♂ (Hastriter and Mendez 2000).

###### Remarks.

According to the revision of the genus *Hectopsylla* by Hastriter and Mendez (2000), H. (H.) cypha can be distinguished from all other species of the genus by the following characters: in males the median lobe presents a lateral patch of long thin setae; in females the dorsal margin of metepimeron is heavily sclerotized and usually with three setae. The host species surely corresponds to *Lagostomus maximus* (Desmarest), because this is the only extant genus. Zapallar is in the Monte Desert of Mountains and Isolated Valleys eco-region.

##### Hectopsylla (Hectopsylla) gracilis

Taxon classificationAnimaliaSiphonapteraTungidae

Mahnert

###### Distribution in Argentina.

Chubut, Jujuy, La Rioja, Mendoza, Neuquén, Río Negro, and Salta (López Berrizbeitia et al. 2013; Lareschi et al. 2016).

###### Material examined.

Arauco Department: (3) 2 km S Río de La Punta on provincial road 7 (28°45'28.8"S; 66°47'09.3"W), 996 m, *G. chacoensis*, 1.IX.2014, RTS (75), 1 ♂ CMLA (600). Castro Barros Department: (7) Anillaco, behind cemetery (28°48'49.04"S, 66°56'0.75"W), 1365 m *Eligmodontia moreni* (Thomas), 10.IX.2014, RTS (77), 2 ♀ CMLA (601, 602); (8) 800 m E Anillaco (28°48.572’ S; 66°55.193’ W), 780 m, *Andalgalomys olrogi* Williams and Mares,17.IV.2012,CML (9747), 4 ♀ CMLA (594, 595, 596, 597); *G. chacoensis*, 17.IV.2012, CML (9748), 1 ♀ CMLA (593); *Eligmodontia typus* F. Cuvier, 18.IV.2012, CML (9751), 2 ♀ CMLA (598, 599) (López Berrizbeitia et al. 2013).

###### Remarks.

This species can be distinguished from all other species of the genus by the following characters: in males, the median lobe of distal portion of sternum IX is enlarged with a concavity at the ventral margin, the apical margin of process of clasper is straight; in females the hilla is much narrower than width of bulga, the duct of spermatheca is connecting at cribriform area of bulga on ventral apical margin and the base of metatarsal claw do not present a sinus (see Hastriter and Mendez 2000). Hectopsylla (Hectopsylla) gracilis infesting *E. moreni* constitutes a new flea-host association; this result was expected since *H.* (*H*) *gracilis* has been collected on *Eligmodontia hirtipes* and *E. typus* (Lareschi et al. 2016). All localities correspond to the Monte Desert of Mountains and Isolated Valleys eco-region.

### Familia Stephanocircidae

#### Subfamily Craneopsyllinae

##### Craneopsylla minerva

Taxon classificationAnimaliaSiphonapteraStephanocircidae

(Rothschild)

Fig. 2a

###### Distribution in Argentina.

Buenos Aires, Catamarca, Chubut, Córdoba, Jujuy, La Pampa, Mendoza, Neuquén, Río Negro, Salta, Santa Fe, Santiago del Estero, Tierra del Fuego, and Tucumán (Lareschi et al. 2016).

**Figure 2.**
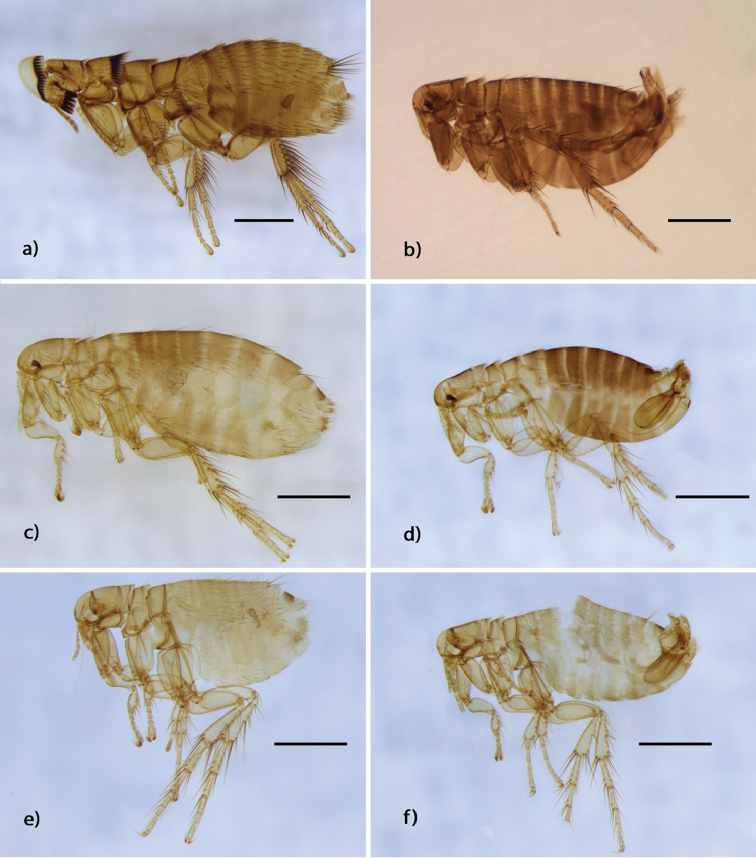
Species of fleas representing the first record for La Rioja Province, Argentina. **a** *Craneopsylla minerva*, ♀ CMLA (608) (Stephanocircidae **b** Polygenis (Polygenis) acodontis, ♂ CMLA (674) (Rhopalopsyllidae) **c** *Delostichus talis*, ♀ CMLA (616) (Rhopalopsyllidae) **d** *Delostichus talis*, ♂ CMLA (619) (Rhopalopsyllidae) **e** *Tiamastus palpalis*, ♀ CMLA (631) (Rhopalopsyllidae) **f** *Tiamastus palpalis*, ♂ CMLA (629) (Rhopalopsyllidae). Scale=500 um.

###### Material examined.

Famatina Department: (10) 5 km S of Cañón del Ocre (28°51'55.9"S, 67°41'26.3"W), 2495 m, *Phyllotis xanthopygus*, 6.XI.2013, RTS (40), 1 ♀ CMLA (608). General Facundo J. Quiroga Department: (14) 2 km E of Malanzán, camping El Descanso (30°48'37.7"S, 66°34'40.3"W), 957 m, *G. chacoensis*, 10.XI.2014, RTS (129), 1 ♀ CMLA (611). San Martín Department: (15) Ulapes, 1 km W of plaza principal de Ulapes (31°34'35"S, 66°14'55"W), 493 m, *G. chacoensis*, 4.X.2014, RTS (84), 2 ♀ CMLA (609, 610).

###### Additional records.

Capital Department: (9) Cuesta La Cébila, 22 km NW of Chumbicha, by route 60 (28°50'S, 66°24'W), 1066 m *Akodon simulator* Thomas, CML (3752), 1 ♀ (Lareschi et al. 2003).

###### Remarks.

The genus *Craneopsylla* is monotypic, and *C. minerva* is distinguished mainly by the genal bristles on the level of the proximal portion of the mouthparts and adjacent structures (Hopkins and Rothschild 1956). Although some authors (Hopkins 1951; Lareschi et al. 2016) consider there are two subspecies, *C. m. minerva* (Rothschild) and *C. m. wolffhuegeli* (Rothschild), Del Ponte (1977) considered *C. minerva* and *C. wolffhuegeli* to be valid species. We believe more detailed morphological and molecular studies are needed to resolve this taxonomic issue. Here we accept these taxa at the species level. Lareschi et al. (2003) cited as *Akodon simulator* Thomas the name of the host species, but we use *A. glaucinus* following Coyner et al. (2013). The authors cited the collecting locality as belonging to Catamarca Province but, in effect, it is placed within La Rioja Province. Moreover, in the same study, a specimen cited as *C. minerva*, was reidentified by us as *Polygenis acodontis*, a member of another family, Rhopalopsyllidae (see below). All localities cited in Material Examined and Additional Records correspond to the Dry Chaco and Monte Desert of Mountains and Isolated Valleys eco-regions.

### Family Rhopalopsyllidae

#### Subfamily Parapsyllinae

##### Delostichus talis

Taxon classificationAnimaliaSiphonapteraRhopalopsyllidae

(Jordan)

Fig. 2c, d

###### Distribution in Argentina.

Buenos Aires, Chubut, La Pampa, Mendoza, Río Negro, and San Juan (Lareschi et al. 2016).

###### Material examined.

Vinchina Department: (2) Quebrada de Santo Domingo 30 km SW of Jagüé (28°31'34.7"S, 68°46'13.8"W), 3131 m *Microcavia* sp., 6.III.2015, RTS (212), 3 ♂ CMLA (619, 620, 621), 6 ♀ CMLA (613, 614, 615, 616, 617, 618).

###### Remarks.

*Delostichus talis* differs from the other species of the genus by the distal arm of sternum IX which is widest at the middle and have a pointed apex in males; and the posterior margin of sternum VII presents a broad lobe in females (Smit 1987). This is the first record of the genus *Delostichus* for La Rioja Province and the first record of the species *D. talis* for northwestern Argentina. The geographical distribution of the species is extended~ 400 km north of the northernmost available record, cited by Lareschi et al. (2016) (Las Casuarinas, San Juan Province). The locality of the Material Examined corresponds to the Puna eco-region.

#### Subfamily Rhopalopsyllinae

##### Polygenis (Polygenis) acodontis

Taxon classificationAnimaliaSiphonapteraRhopalopsyllidae

(Jordan & Rothschild)

Fig. 2b

###### Distribution in Argentina.

Buenos Aires, Catamarca, Córdoba, Jujuy, La Pampa, Salta, and Tucumán (Lareschi et al. 2016).

###### Material examined.

Capital Department: (9) Cuesta La Cébila, 22 km NW of Chumbicha, by route 60 (28°50'S, 66°24'W), 1066 m *Akodon glaucinus*, 25.IX.1993, CML (3751), 1 ♀ CMLA (674).

###### Remarks.

The main morphological characters that distinguish *P. acodontis* from the other known species of genus are: in males, the distal arm of sternum IX with a dense fringe of setae along its ventral margin and sternum VIII is strongly emarginated ventro-apically; in females the posterior margin of sternum VII presents a drawn-out median lobe (Smit, 1987). This is the first record of this species for La Rioja Province. The locality of the Material Examined corresponds to the Monte Desert of Mountains and Isolated Valleys eco-region.

##### Polygenis (Polygenis) platensis

Taxon classificationAnimaliaSiphonapteraRhopalopsyllidae

(Jordan & Rothschild)

###### Distribution in Argentina.

Buenos Aires, Córdoba, Chubut, Entre Ríos, Jujuy, La Pampa, La Rioja, Mendoza, Río Negro, Salta, San Luis, San Juan, Santa Cruz, and Santiago del Estero (Lareschi et al. 2016).

###### Material examined.

Castro Barros Department: (4) Reserva Aguada de las Alturas, 4 km W of Anillaco (28°47.942'S, 66°59.749'W), 1188 m, *A. glaucinus*, 19.IV.2012, RTS (19), 1 ♂ CMLA (605) (López Berrizbeitia et al. 2013). San Martín Department: (15) Ulapes, 1 W of plaza principal de Ulapes (31°34'35"S, 66°14'55"W), 493 m, *G. chacoensis*, 4.X.2014, RTS (84), 2 ♀ CMLA (622, 623); RTS (85), 1 ♀ CMLA (624); RTS (93), 2 ♂ CMLA (625,626), 2 ♀ CMLA (627, 628).

###### Additional records.

“Guayapa 30.59"S, 65.59 W", Order Rodentia (unknown species) (Smit 1987).

###### Remarks.

Males of *P. platensis* are distinguished by the narrow distal arm of sternum IX with scattered lateral setae and by the posterior margin of sternum VIII which presents a right angle. Females have the ventral margin of bulga of spermatheca markedly indented (Smit 1987); however, Lareschi and Linardi (2009) observed a variation on this character and they reported that spermatheca can be indented or not. With respect to the record from “Guayapa”, we found that the coordinates given by Smit (1987) place the collecting locality at “app. 3 km N of El Milagro, General Ocampo Department”. The locality “Guayapa” in La Rioja Province is located at 29°51'41.18"S, 67°14'22.99"W, which is about 180 km SE of the previous coordinates. López Berrizbeitia et al. (2013) cited the host as *Akodon simulator*, but we now use the name *A. glaucinus* following Coyner et al. (2013). The collection from Ulapes from *G. chacoensis* constitutes a new flea-host association, this result was expected, because P. (P.) platensis has been recorded on *Graomys griseoflavus* (Lareschi et al. 2016). The localities recorded are from the Dry Chaco and Monte Desert of Mountains and Isolated Valleys eco-regions.

##### Polygenis (Polygenis) rimatus

Taxon classificationAnimaliaSiphonapteraRhopalopsyllidae

(Jordan)

###### Distribution in Argentina.

Buenos Aires, Chubut, Formosa, La Rioja, Misiones, Neuquén, San Juan, and Santiago del Estero (Lareschi et al. 2016).

###### Material examined.

None.

###### Additional records.

No specific locality, *Cavia aperea* Erxleben, 1 ♀ (Smit 1987).

###### Remarks.

This species can be distinguished from the other species of *Polygenis* by the labial palp reaching the apex of fore coxa, the acetabular seta is below level of the upper margin of acetabulum in males; the posterior margin of sternum VII in females with a smaller lobe (Smit 1987). The host was probably erroneously identified because *C. aperea*, a member of the family Caviidae, is not known to occur in La Rioja Province (Patton et al. 2015). The only members of this family known to be present in La Rioja are *Galea leucoblephara* and *Microcavia australis*.

##### Tiamastus palpalis

Taxon classificationAnimaliaSiphonapteraRhopalopsyllidae

(Rothschild)

Fig. 2e, f

###### Distribution in Argentina.

Catamarca, Jujuy, La Pampa, Mendoza, Santa Fe, Santiago del Estero, and Tucumán (Lareschi et al. 2016).

###### Material examined.

San Blas Department: (1) 700 m E of National Route 40 (28°24'17.4"S, 67°04'48.4"W), 1123 m, *Ctenomys* sp., 29.II.2016, RTS (300), 1 ♀ CMLA (631). Castro Barros Department: (5) Anillaco, 500 m W of plaza de Anillaco (28°48'40.30"S, 66°55'55.55"W), 500 m, *Ctenomys* sp., IX.2015, released, ♂ CMLA (629), 5 ♀ CMLA (630, 632, 633, 634, 635).

###### Remarks.

This species can be distinguished from all other species of the genus by the following characters: apical half of telomere narrowing to a sharp apex in males; in females, the posterior margin of sternum VII with a narrow sinus and spermatheca with bulga as long as hilla (Smit 1987). These are the first records of the species for La Rioja Province. The localities correspond to the Monte Desert of Mountains and Isolated Valleys eco-region.

### Family Ctenophthalmidae

#### Subfamily Ctenophthalminae

##### *Neotyphloceras crassispina* Rothschild

###### Neotyphloceras crassispina hemisus

Taxon classificationAnimaliaSiphonapteraCtenophthalmidae

Jordan

####### Distribution in Argentina.

Catamarca, Jujuy, La Rioja, Mendoza, Neuquén, Río Negro, and Salta (López Berrizbeitia et al. 2013; Lareschi et al. 2016).

####### Material examined.

Castro Barros Department: (4) Reserva Aguada de las Alturas, 4 km W Anillaco (28°47.942'S, 66°59.749'W), 1188 m, *Phyllotis xanthopygus* (Waterhouse), 18.IV.2012, CML (9753), 2 ♂ CMLA (603, 604) (López Berrizbeitia et al. 2013). Famatina Department: (11) 8 km NE of Cañón del Ocre, (28°57'37.3"S, 67°41'26.3"W), 3127 m, *P. xanthopygus*, 6.XI.2013, RTS (39), 2 ♀ CMLA (606, 607).

####### Additional records.

Coronel Felipe Varela Department: (13) 1 km N Los Tambillos (29°22'S, 67°47'W), 1951 m, *Graomys griseoflavus* (J. A. Allen), 30.IX.1993, CML (9400), 1 ♂, 1 ♀ (Lareschi et al. 2003).

####### Remarks.

*Neotyphloceras crassispina hemisus* differs from all other species and subspecies of the genus by the presence of abdominal spinelets; females are unique by the apical margin of sternum VII wide, and the contour of the distal margin rounded or slightly convex in the lower portion; males differ by a combination of characters in the genitalia (López Berrizbeitia et al. 2015). Lareschi et al. (2003) cited *G. griseoflavus* as the name of the host for this species, but now it should be treated as *G. chacoensis*, following Braun and Patton (2015); it is also important to note that the correct name for the locality is “Los Tambillos,” and not “Los Tombillos,” as cited by these authors. All localities correspond to the Monte Desert of Mountains and Isolated Valleys eco-region.

### Family Pulicidae

#### Subfamily Pulicinae

##### Pulex irritans

Taxon classificationAnimaliaSiphonapteraPulicidae

Linnaeus

###### Distribution in Argentina.

Buenos Aires, Catamarca, Córdoba, Chubut, Formosa, La Rioja, Mendoza, Neuquén, Río Negro, Salta, San Luis, Santiago del Estero, and Tucumán (Lareschi et al. 2016).

###### Material examined.

None.

###### Additional records.

“Between Olta and Santa Rita de Catuna” *Dolichotis patagonica* (Zimmermann), 1 ♀; *Lepus* sp., 1 ♀ (Hopkins and Rothschild 1953).

###### Remarks.

*Pulex irritans* is distinguished by the disposition of the internal incrassation of frons, which is hardly projected inwards from margin of frons (Hopkins and Rothschild 1953). Hopkins and Rothschild (1953) cited *Dolichotis patagonica* as the host species, but the correct spelling is *D. patagonum*. They also cited *Lepus* sp. as a host, which corresponds to *Lepus europaeus* Pallas, because this is the only *Lepus* sp. found in Argentina. Because the authors did not cite an exact locality, we only indicate the coordinates for the two main localities cited by them: Olta 30°37'53.26"S, 66°15'48.87"W; Santa Rita de Catuna 30°57'03.87"S, 66°13'02.48"W.

#### Subfamily Xenopsyllinae

##### Xenopsylla cheopis

Taxon classificationAnimaliaSiphonapteraPulicidae

(Rothschild)

###### Distribution in Argentina.

Buenos Aires and La Rioja (Lareschi et al. 2016).

###### Material examined.

None.

###### Additional records.

No specific locality, *Epimys decumanus* Pallas, 1 ♂; *Epimys* sp., 4 ♂, many ♀ (Hopkins and Rothschild 1953).

###### Remarks.

*Xenopsylla cheopis* differs from all other species of the genus by the following characters: in males, sternum IX with distal arm equally sclerotized throughout; in females, the tail of spermatheca is not strongly swollen (Johnson 1957). Hopkins and Rothschild (1953) cited *Epimys decumanus* Pallas and *Epimys* sp. as hosts which, follow the nomenclature of Wilson and Reeder (2005), but are synonymous with *R. norvegicus* Berkenhout and *Rattus* sp., respectively. *Xenopsylla cheopis* is the most efficient vector of *Yersinia pestis* (Lehmann and Neumann), responsible for the bubonic plague (Boyer et al. 2014). Although only old records of this species are cited from La Rioja Province, it is important to mention that this is the only record for northwestern Argentina. The absence of more and newer records of this species is probably a reflection of mammalogists exerting little effort in the study of domestic rats.

### Family Ischnopsyllidae

#### Myodopsylla isidori

Taxon classificationAnimaliaSiphonapteraIschnopsyllidae

(Weyenbergh)

##### Distribution in Argentina.

Buenos Aires, Catamarca, Córdoba, Corrientes, Entre Ríos, Jujuy, La Rioja, Neuquén, Río Negro, San Luis, Salta, Santiago del Estero, and Tucumán (Lareschi et al. 2016).

##### Material examined.

None.

##### Additional records.

No specific locality, Order Chiroptera (unknown species), 1 ♂, 1 ♀ (Hopkins and Rothschild 1956).

##### Remarks.

This species is distinguishable from the other known species of *Myodopsylla* by the movable process with a proximal apical angle of about 60°; females with the frons oblique (Hopkins and Rothschild 1956). *Myodopsylla isidori* is restricted to South America (Hopkins and Rothschild 1956) and parasitizes bats of the families Molossidae and Vespertilionidae (Autino et al. 2009). Collection of bats and their ectoparasites, particularly in shelters, is needed in the province of La Rioja to elucidate the bat species diversity and their associated fleas.

#### Myodopsylla

Taxon classificationAnimaliaSiphonapteraIschnopsyllidae

sp.

##### Material examined.

Castro Barros Department: (6) Anillaco 1.7 m E of CRILAR (28°48'46.00"S, 66°55'50.44"W), 1357 m *Myotis dinellii*, 12.XI.2015, released specimen, 1 ♀ CMLA (612).

##### Remarks.

This is the second record for this undetermined species of *Myodopsylla* for La Rioja Province. More specimens, males and females, and comparisons with material deposited in collections are necessary to identify the species with confidence. The collecting locality corresponds to the Monte Desert of Mountains and Isolated Valleys eco-region.

### Key yo identification of fleas from La Rioja Province, modified from Hopkins and Rothschild (1953, 1956), Smit (1987), and Hastriter and Mendez (2000)

**Table:** 

1	Genal comb present	**2**
–	Genal comb absent	**5**
2	Anterior helmet-comb present; five to eight spines in the genal comb	Family Stephanocircidae; Subfamily Craneopsyllinae, ***Craneopsylla minerva***
–	Anterior helmet-comb absent; two or four spines in the genal comb	**3**
3	Genal comb composed of four spines	Family Ctenophthalmidae; Subfamily Ctenophthalminae; ***Neotyphloceras crassispina hemisus***
–	Genal comb composed of two spines	Family Ischnopsyllidae, Subfamily Ischnopsyllinae; Genus *Myodopsylla...**4***
4	Males with proximal apical angle of movable process about 60°; females with frons markedly oblique	***Myodopsylla isidori***
–	Males unknown; females with frons much more convex	***Myodopsylla* sp.**
5	Frontal tubercle present and well developed	Family Rhopalopsyllidae...**6**
–	Frontal tubercle absent	**10**
6	Postantennal region of head mostly with one row of setae	Subfamily Parapsyllinae; ***Delostichus talis***
–	Postantennal region of head with three rows of setae (rarely two)	Subfamily Rhopalopsyllinae...**7**
7	Labial palp extending to or beyond apex of fore trochanter	***Tiamastus palpalis***
–	Labial palp not extending beyond base of fore trochanter	Genus Polygenis (P.)...**8**
9	Males with posterior margin of sternum VIII forming a right angle; females with ventral margin of bulga of spermatheca markedly indented	**Polygenis (P.) platensis**
–	Males with posterior margin of sternum VIII not forming a right angle; females with ventral margin of bulga of spermatheca without distinct indentation	**9**
9	Males with sternum VIII ventro-apically strongly emarginate; females with sternum VII with a protruding median lobe	**Polygenis (P.) acodontis**
–	Males with sternum VIII not emarginate; females with sternum VII with a not protruding median lobe	**Polygenis (P.) rimatus**
10	Inner side of hind coxa with spiniform bristles	Family Pulicidae...**11**
–	Inner side of hind coxa without spiniform bristles	Family Tungidae, Subfamily Tunginae; Genus Hectopsylla (H.)...**12**
11	Pleural rod of mesothorax absent	Subfamily Pulicinae; ***Pulex irritans***
–	Pleural rod of mesothorax present	Subfamily Xenopsyllinae; ***Xenopsylla cheopis***
12	Males with median dorsal lobe of aedeagus well developed; females with dorsal margin of metepimeron heavily sclerotized, usually with three setae	**Hectopsylla (H.) cypha**
–	Males with median dorsal lobe of aedeagus poorly developed; females with dorsal margin of metepimeron not noticeably sclerotized, usually four setae	**Hectopsylla (H.) gracilis**

## Discussion

These results are a contribution to the knowledge of the flea fauna of La Rioja, a neglected province regarding the study of mammals and their parasites; the last study about ectoparasites from La Rioja Province was published by López Berrizbeitia et al. (2013), reporting new records of fleas and mites for the province; therefore, here, the number of hosts and fleas is increased, allowing us to have a better representation of the flea-host associations. Thirteen species, nine genera, and six families of fleas are recorded for La Rioja Province. *Craneopsylla minerva*, *Delostichus talis*, *Polygenis acodontis*, and *Tiamastus palpalis* represent the first records for these species for La Rioja Province.

The occurrence of *C. minerva* was expected because it is distributed in some neighboring provinces of La Rioja (Lareschi et al. 2016). The distribution of *D. talis* is extended approximately 400 km to the north. *Delostichus* is recorded for the first time for northwestern Argentina, infesting *Microcavia*. Smit (1987) considered the caviids, *Galea leucoblephara* and *Microcavia australis*, to be the primary hosts of *D. talis*. *Tiamastus palpalis* is also associated mainly with caviid rodents (Smit 1987); we found it infesting *Ctenomys* spp., coincident with previous reports. This species of the flea was recorded on *Ctenomys haigi* and *Ctenomys juris* from Jujuy Province (Johnson 1957; Smit 1987) and *Ctenomys andersoni* from Bolivia (Pucu et al. 2014). Out of 13 species recorded, two are endemic to Argentina: *D. talis* and H. (H.) gracilis (Lareschi et al. 2016).

Most species of fleas reported in the current study inhabit the Monte Desert of Mountains and Isolated Valleys ecoregion, and two are also found in the Dry Chaco eco-region, *Craneopsylla minerva* and *Polygenis platensis*. These two species showed the highest prevalence and mean abundance on small mammals in a study carried out in the Monte Desert biome (Lareschi et al. 2004). *Delostichus talis* was recorded exclusively in the Puna eco-region, where the vegetation is dominated by shrub steppe, characterized by scattered shrubs and stony or saline soils covered by sparse vegetation. In Argentina, the Puna is located in the north and extends from Jujuy Province to north of San Juan Province (Burkart et al. 1999). This is the first record of *D. talis* for Puna eco-region. The previous northernmost known record in the San Juan Province (Las Casuarinas) (Lareschi et al. 2016) corresponds to Monte Desert of Mountains and Isolated Valleys eco-region.

Knowledge of the distribution and hosts of *Delostichus* spp. is important because they are potential vectors of the agent of bubonic plague in the Chile-Andean subregion (Macchiavello 1948; Gimenez et al. 1964; Beaucournu et al. 2013). Likewise, *Xenopsylla cheopis* is a competent vector of *Yersinia pestis*, but unlike *D. talis*, this species infests introduced rodents (*Rattus* spp.). *Craneopsylla minerva* was recorded harboring this pathogen in Ayabaca Province, Piura, Perú (Pozo et al., 2005). Additional research is needed to determine whether these species are vectors in Argentina.

The nomenclature of the hosts was updated according to the current taxonomy and distribution, although identifications of some species are in process. The correct identification of hosts is fundamental to avoid misinterpretations about parasite-host associations (Robles 2010). Research on identity of host species has long been neglected by parasitologists. We emphasize the importance of joint research between parasitologists and mammalogists to insure proper identification of both parasites and their hosts. Because these parasites are potentially important as vectors of infectious agents causing human and animal disease (Hastriter and Whiting 2003), knowledge of their hosts is of fundamental zoological and epidemiological importance, especially in matters of public health.

Some regions as the Puna and High Andes ecoregions in the Province La Rioja have not yet been adequately sampled for ectoparasites, particularly those areas where some species are potentially present, and where specimens are needed to resolve taxonomic conflicts.

## Supplementary Material

XML Treatment for Hectopsylla (Hectopsylla) cypha

XML Treatment for Hectopsylla (Hectopsylla) gracilis

XML Treatment for Craneopsylla minerva

XML Treatment for Delostichus talis

XML Treatment for Polygenis (Polygenis) acodontis

XML Treatment for Polygenis (Polygenis) platensis

XML Treatment for Polygenis (Polygenis) rimatus

XML Treatment for Tiamastus palpalis

XML Treatment for Neotyphloceras crassispina hemisus

XML Treatment for Pulex irritans

XML Treatment for Xenopsylla cheopis

XML Treatment for Myodopsylla isidori

XML Treatment for Myodopsylla
